# Medicare Advantage Plan Star Ratings and County Social Vulnerability

**DOI:** 10.1001/jamanetworkopen.2024.24089

**Published:** 2024-07-23

**Authors:** Avni Gupta, Diana Silver, David J. Meyers, Sherry Glied, José A. Pagán

**Affiliations:** 1 Department of Public Health Policy and Management, School of Global Public Health, New York University, New York; 2 Healthcare Coverage and Access, The Commonwealth Fund, New York, New York; 3 Department of Health Services, Policy & Practice, Brown University School of Public Health, Providence, Rhode Island; 4 Robert F. Wagner Graduate School of Public Service, New York University, New York

## Abstract

**Importance:**

The star rating of a Medicare Advantage (MA) plan is meant to represent plan performance, and it determines the size of quality bonuses. Consumer access to MA plans with a high star rating may vary by the extent of social vulnerability in geographic regions.

**Objective:**

To examine the association between a county’s Social Vulnerability Index (SVI) and the star rating of a county’s MA plans.

**Design, Setting, and Participants:**

This cross-sectional study used 2023 Centers for Medicare & Medicaid Services data for all MA plans linked to 2020 county-level SVI data from the Centers for Disease Control and Prevention. Data were analyzed from March to October 2023.

**Exposure:**

Quintile rank of county based on composite and theme-specific SVI scores, with quartile 1 (Q1) representing the least vulnerable counties and Q5, the most vulnerable counties. The SVI is a multidimensional measure of a county’s social vulnerability across 4 themes: socioeconomic status, household characteristics (such as disability, age, and language), racial and ethnic minority status, and housing type and transportation.

**Main Outcomes and Measures:**

County-level mean star rating and the number of MA plans with low-rated (<3.5 stars), high-rated (3.5 or 4.0 stars), and highest-rated (≥4.5 stars) plans.

**Results:**

Across 3075 counties, the median county-level star rating was 4.1 (IQR, 3.9-4.3) in Q1 counties and 3.8 (IQR, 3.6-4.0) in Q5 counties (*P* < .001). The mean star rating of MA plans was lower (difference, −0.24 points; 95% CI, −0.28 to −0.21 points; *P* < .001), the number of low-rated plans was higher (incidence rate ratio, 1.81; 95% CI, 1.61-2.06; *P* < .001), and the number of highest-rated plans was lower (incidence rate ratio, 0.75; 95% CI, 0.70-0.81; *P* < .001) in Q5 counties compared with Q1 counties. Similar patterns were found across theme-specific SVI score quintiles and for 2022 star ratings.

**Conclusions and Relevance:**

In this cross-sectional study, the most socially vulnerable counties were found to have the fewest highest-rated plans for MA beneficiaries. As MA enrollment grows in socially vulnerable regions, this may exacerbate regional differences in health outcomes for Medicare beneficiaries.

## Key Points

**Question:**

Do the star ratings of Medicare Advantage (MA) plans vary by the social vulnerability of a county?

**Findings:**

In this cross-sectional study of MA plans in 3075 US counties in 2023, the mean star rating and the number of plans with the highest star rating were lower in counties with a higher Social Vulnerability Index score.

**Meaning:**

The findings suggest that Medicare beneficiaries living in counties with higher social vulnerability have fewer highly rated MA plans available in their county, which may exacerbate regional disparities in health outcomes.

## Teaser

This cross-sectional study examines county-level variation in star ratings of Medicare Advantage plans by measures of social vulnerability.

## Introduction

More than half of all Medicare beneficiaries are now enrolled in Medicare Advantage (MA) plans.^1^ The Centers for Medicare & Medicaid Services (CMS) uses a 5-star rating system to measure the quality of these private plans across nearly 40 indicators, spanning domains such as preventive care, chronic disease management, patient experience, customer complaints, customer service, and drug safety and pricing.^2^ The CMS integrates data to generate the ratings from a comprehensive set of sources, such as the Medicare Consumer Assessment of Healthcare Providers & Systems survey, the Healthcare Effectiveness Data and Information Set, the Health Outcomes Survey, CMS administrative data, and the Health Plan Management System.^2^ The CMS publishes star ratings at the level of MA contracts, and each plan in a contract is assigned the same rating.

Star ratings are supposed to capture the performance of an MA plan during previous years. While the evidence on the association of star ratings with outcomes is mixed,^3,4^ star ratings have been shown to affect beneficiary enrollment, and MA beneficiaries are more likely to enroll in plans with higher star ratings.^5^ Higher enrollment increases top-line revenue. High-star plans also receive higher rebates and quality bonuses, leading to potentially greater resources with high-rated plans for investing in better care infrastructure and for funding non-Medicare benefits, including lower Part B premiums, reduced out-of-pocket spending, and/or supplemental benefits such as dental, vision, hearing, fitness, and meals at no extra or low costs to beneficiaries.^6,7^

Given that socioeconomically disadvantaged and racial and ethnic minority beneficiaries have less health care access and utilization than their more advantaged peers,^8,9,10^ it is possible that MA plans serving a higher proportion of such beneficiaries have lower star ratings. Racial and ethnic disparities in hospitalizations for ambulatory care–sensitive conditions have been found to be higher in low-rated vs high-rated MA plans.^11^ This could suggest that low-rated plans might be lacking the resources or priorities to invest in quality or equity, leading to widened disparities.

To date, however, there is limited understanding of the association between MA plan star ratings and the level of social vulnerability of enrollees. Park et al^12^ found that racial and ethnic minority enrollees had on average a lower number of high-rated plans (≥4.0 stars) and a higher number of low-rated plans (≤3.5 stars) in their counties of residence. Park et al^11^ also found that lower enrollment of racial and ethnic minority enrollees in plans rated 4.0 to 4.5 stars compared with White enrollees was in part associated with the lower availability of high-rated plans in their counties of residence.

The goal of this study was to examine geographic variation in MA plan star ratings by comprehensive measures of county-level social vulnerability. Characterizing such geographic variation is important because beneficiaries living in low-income areas and with higher neighborhood disadvantage are more likely to enroll in MA than in traditional Medicare.^13^ In addition, the largest growth in MA enrollment in recent years has been among beneficiaries living in areas with higher socioeconomic neighborhood disadvantage.^13^ If star ratings of MA plans vary with area-level social vulnerability, such geographic variation patterns may lead to access barriers for some Medicare beneficiaries and fewer opportunities to realize the potential benefits of receiving MA coverage through a plan with a high star rating. Such variations in star rating by area-level social vulnerability could suggest that plans serving vulnerable regions might need additional support to better serve their population. As CMS strengthens its focus on equity, this knowledge could inform policies to promote better health care access and higher health care quality among Medicare beneficiaries.^14,15^

## Methods

### Databases

We conducted a cross-sectional study using publicly available data sources on MA plans and counties; thus, informed consent was not applicable and the study was deemed exempt from institutional review board approval by New York University. This study followed the Strengthening the Reporting of Observational Studies in Epidemiology (STROBE) reporting guideline for cross-sectional studies.

We constructed a county-level dataset that merged the CMS MA Landscape, Service Area, and Star Ratings files from 2023 with Social Vulnerability Index (SVI) data from 2020, the most recent year of SVI data available. We included all MA plans in the landscape file with a star rating, contract, and plan identification number to allow linkage across MA files. The SVI is a multidimensional composite measure of area-level social vulnerability developed by the Centers for Disease Control and Prevention (CDC) to identify areas with the least resources and infrastructure, because such areas are most at risk during any disaster and, thus, need the most support before, during, and after a hazardous event.^16,17^ Recently, the SVI has been shown to be associated with several health care measures, such as health care access,^18^ the availability of supplemental benefits in MA,^19^ the availability of home health services,^20^ and cancer screening rates.^16^ The SVI data were obtained from the CDC Agency for Toxic Substances and Disease Registry website. The SVI is constructed by the CDC using US census data to rank each county on 16 social determinants of health spanning 4 themes: socioeconomic status (poverty, unemployment, housing cost burden, high school diploma status, and health insurance), household characteristics (disability status, household members aged ≥65 and ≤17 years, single-parent households, and English language proficiency), racial and ethnic minority status (minoritized racial and ethnic group: American Indian or Alaska Native, Asian, Black or African American, Native Hawaiian or Other Pacific Islander, other, and multiracial), and housing type and transportation (multiunit structure, mobile home, crowding, no vehicle, and group quarters). Theme-specific scores and the composite score indicate the relative social vulnerability of a particular county on a scale from 0 to 1, where 0 represents least social vulnerability. Analyses were conducted from March to October 2023.

### Measures

We included 5 outcome measures at the county level using the overall Part C and D star rating in the respective year: mean star rating of MA plans, number of low-star (<3.5 stars) plans, number of high-star (3.5 or 4.0 stars) plans, number of highest-star (4.5 or 5.0 stars) plans, and presence of at least 1 plan rated 5.0 stars. The exposure variables were quintiles of the composite SVI score and of each of the 4 themes constituting the SVI.^16,21,22^ The SVI quintiles were delineated as less than 0.2000, 0.2000 to 0.4001, 0.4004 to 0.5900, 0.6000 to 0.8001, and 0.8004 to 1.000. Counties in quintile 1 (Q1)—that is, those with an SVI from 0 to less than 0.2000—served as the reference group and hereafter will be referred to as counties with the lowest or least vulnerability. These counties were compared with those in Q2 (low vulnerability), Q3 (moderate vulnerability), Q4 (high vulnerability), and Q5 (highest vulnerability).

### Statistical Analysis

We calculated summary statistics for outcome measures (ie, star rating measures) stratified by SVI categories and report the medians and IQRs since the Shapiro-Wilk normality test rejected the hypothesis of normal distribution of our outcomes. The Kruskal-Wallis test was then used to identify statistically significant differences in an outcome across SVI categories.^23^ We then used linear regression to estimate the difference in mean star ratings between counties in Q2 to Q5 compared with counties in Q1. We used negative binomial regression to examine the incidence rate ratio (IRR) of the number of high-, highest-, and low-star plans between counties in Q2 to Q5 compared with counties in Q1. We used logistic regression to examine the odds of having at least 1 plan rated 5.0 stars in Q2 to Q5 counties compared with Q1 counties. For the composite SVI score, we estimated the margins (along with 95% CIs), which can be interpreted as mean of the mean star ratings in counties in an SVI quintile category. For the 4 themes, we calculated the marginal effect size for the association of each vulnerability theme with each outcome. Marginal effect sizes can be interpreted as the difference in the outcome measure between SVI Q2 to Q5 and Q1. We used mapping tools to show the geographic distribution of the mean star rating, categorized as less than 3.5 stars, 3.5 to 4.0 stars, or 4.5 stars or greater, and the composite SVI score quintiles (eFigure 2 in Supplement 1). Because star rating calculations for 2022 were affected by changes implemented by CMS to account for the impact of the COVID-19 pandemic, we conducted a sensitivity analysis using data from 2022 as a robustness check on our findings. We conducted another sensitivity analysis using the Social Deprivation Index. All analyses were conducted in Stata/SE, version 17.0 (StataCorp LLC). Statistical significance was set at *P* < .05, and hypothesis tests were 2-sided.

## Results

The 2023 MA Landscape file contained 90 507 plans across 3126 unique counties. We excluded 51 counties where no MA plan offered in 2023 had a star rating for 2023, either because the plans lacked sufficient data to measure the star rating or plans were too new to be assigned a star rating (eFigure 1 and eTable 1 in Supplement 1). Among the remaining 3075 counties, the number of counties across each quintile were 620 in Q1, 618 in Q2, 608 in Q3, 617 in Q4, and 612 in Q5. The median county-level star rating was 4.1 (IQR, 3.9-4.3) in Q1 counties (lowest social vulnerability) and 3.8 (IQR, 3.6-4.0) in Q5 counties (highest social vulnerability) (*P* < .001) (Table). The median number of highest-rated plans in a county was highest for Q3 counties (14 [IQR, 7-19]) and lowest for Q5 counties (11 [IQR, 8-15]) (*P* < .001) (Table). However, the median number of low-rated plans in a county was highest for Q5 counties (4 [IQR, 2-8]) and lowest for Q1 counties (2 [IQR, 0-4]) (*P* < .001) (Table). While 208 of 620 counties in Q1 (33.5%) had at least 1 plan rated 5.0 stars, 160 of 612 Q5 counties (26.1%) had at least 1 plan rated 5.0 stars. After Alaska, where the 2 plans offered were both rated 4.5 stars or higher (100%), the 2 states with the highest concentration of highest-rated plans were Wisconsin (96 of 115 plans [83.5%]) and Minnesota (69 of 93 plans [74.2%]). The 2 states with the highest concentration of lowest-rated plans were Connecticut (16 of 45 plans [35.6%]) and Washington (43 of 133 plans [32.3%]) (eFigure 2 in Supplement 1).

**Table.** County-Level Medicare Advantage Plan Star Rating by Quintiles of County-Level Social Vulnerability in 2023

County-level outcome	Median (IQR)						***P* value**
	All counties (n = 3075)	Quintile of composite SVI score					
		Least	Low	Moderate	High	Highest	
Overall SVI score	0.5 (0.2-0.7)	0.1 (0.1-0.2)	0.3 (0.2-0.3)	0.5 (0.5-0.5)	0.7 (0.6-0.7)	0.9 (0.8-1.0)	<.001
Plans, No.							
Overall	29 (19-38)	27 (17-36)	30 (20-40)	31 (20-39)	30 (20-40)	27 (19-35)	<.001
Rated <3.5 stars	4 (0-6)	2 (0-4)	3 (0-6)	4 (1-6)	4 (2-7)	4 (2-8)	<.001
Rated 3.5 or 4.0 stars	10 (6-15)	8 (4-13)	10 (7-15)	11 (7-16)	11 (7-16)	10 (6-15)	<.001
Rated ≥4.5 stars	13 (7-18)	13 (8-21)	13 (7-21)	14 (7-19)	13 (7-17)	11 (8-15)	<.001
Mean star rating	3.9 (3.7-4.1)	4.1 (3.9-4.3)	4.0 (3.8-4.2)	3.9 (3.8-4.1)	3.9 (3.7-4.1)	3.8 (3.6-4.0)	<.001

Abbreviation: SVI, Social Vulnerability Index.

### Composite SVI Score

**Figure 1.**
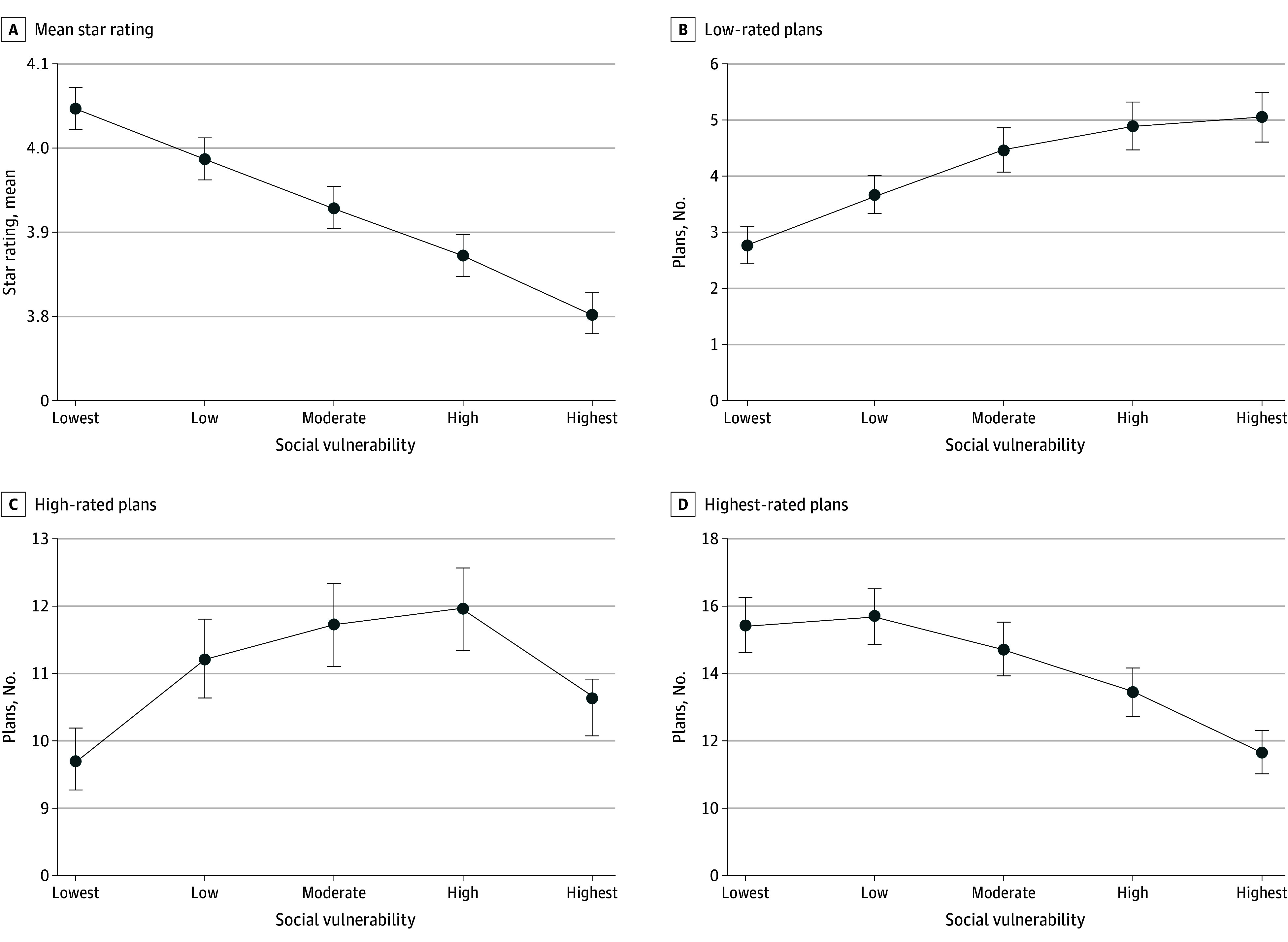
Star Rating Outcomes by Quintiles of the Overall Social Vulnerability Index Score The estimates are margins calculated from 4 bivariable regression models, 1 for each star rating outcome measure, using data from 2023. Margins represent the mean value of an outcome at each quintile of the overall Social Vulnerability Index score. Error bars indicate 95% CIs.

In the regression models, compared with the Q1 counties, the mean star rating based on the composite SVI score was lower in Q5 counties (difference, −0.24 points; 95% CI, −0.28 to −0.21 points; *P* < .001), Q4 counties (−0.17 points; 95% CI, −0.21 to −0.14 points; *P* < .001), Q3 counties (−0.12 points; 95% CI, −0.15 to −0.08 points; *P* < .001), and Q2 counties (−0.06 points; 95% CI, −0.09 to −0.03 points; *P* < .001). The number of low- and high-rated plans was higher in Q2 to Q5 counties compared with Q1 counties (eg, in Q5 counties, the IRR for low-rated plans was 1.81; 95% CI, 1.61-2.06; *P* < .001). However, the number of highest-rated plans was lower in Q5 (IRR, 0.75; 95% CI, 0.70-0.81; *P* < .001) and Q4 (IRR, 0.87; 95% CI, 0.81-0.94; *P* < .001) counties than in Q1 counties (eTable 2 in Supplement 1). Figure 1 shows the mean of each star rating outcome at each SVI quintile. The mean star rating ranged from 4.05 (95% CI, 4.02-4.07) in Q1 counties to 3.80 (95% CI, 3.77-3.83) in Q5 counties. The mean number of low-rated plans was 2.79 (95% CI, 2.53-3.05) in Q1 counties compared with 5.06 (95% CI, 4.62-5.50) in Q5 counties. The number of highest-rated plans was 15.42 (95% CI, 14.61-16.23) in Q1 counties compared with 11.64 (95% CI, 11.01-12.32) in Q5 counties (eTable 3 in Supplement 1). The Q5 counties were less likely than Q1 counties to have at least 1 plan rated 5 stars (−7.4 percentage points; 95% CI, −12.5 to −2.3 percentage points; *P* = .004).

### Theme-Specific Scores and Sensitivity Analysis

**Figure 2.**
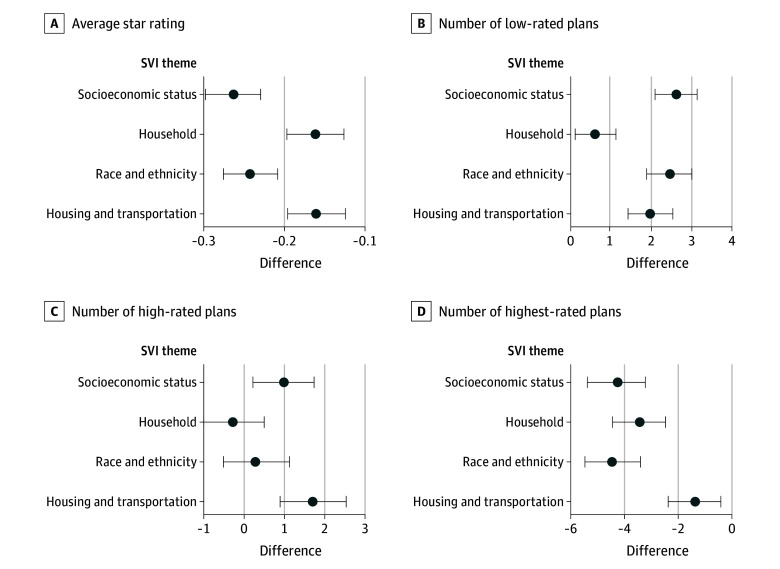
Difference in Star Rating Outcomes in Counties in the Highest vs Lowest Vulnerability Quintile by Overall Social Vulnerability Index (SVI) Themes The estimates shown are marginal effects, which can be interpreted as the difference in the mean star rating outcome between counties in the highest and lowest vulnerability quintiles. Error bars indicate 95% CIs. The analysis was conducted using 2023 star rating data.

For each of the 4 themes of the SVI, the mean star rating was lower, the number of low- and high-rated plans was higher, and the number of highest-rated plans was lower in Q5 counties compared with Q1 counties except for no difference in the number of high-rated plans for the household and race and ethnicity themes (Figure 2; eTable 2 in Supplement 1 shows regression coefficients, and eTable 4 in Supplement 1 shows marginal effect sizes). Counties in Q5 were also less likely to have any 5.0-star rated plan than were Q1 counties based on the socioeconomic status (−9.0 percentage points; 95% CI, −14.1 to −3.9 percentage points; *P* = .001), household (−10.7 percentage points; 95% CI, −15.8 to −5.5 percentage points; *P* = .001), and race and ethnicity (−7.9 percentage points; 95% CI, −13.1 to −2.7 percentage points; *P* = .003) themes of the SVI. Results were similar in 2022 (eTable 5, eFigure 3, and eAppendix in Supplement 1) or when using the Social Deprivation Index (eTable 6 in Supplement 1).

## Discussion

Using data from all MA plans offered in 2023, we found that the plans offered in counties with the highest social vulnerability score were more likely to be rated less than 3.5 stars and less likely to be rated 4.5 stars or higher. This pattern was consistent when the counties were assessed both by their composite SVI score and by each of the 4 themes that made up the composite score. The most vulnerable counties were also less likely than the least vulnerable counties to have a 5.0-star–rated plan. These findings persisted using data from 2022. Although the 2022 star ratings were subject to COVID-19 pandemic–related regulatory flexibilities, which were lifted for 2023 star ratings,^24^ the patterns of star ratings by regional social vulnerability were seen in both 2022 and 2023. We included all types of MA plans with a star rating, making our findings generalizable to the wider MA program.

Our findings suggest that in 2022 and 2023, Medicare-eligible residents of counties with an overall higher burden of disadvantage, including greater incidence of poverty, unemployment, lower educational attainment, no insurance, elderly individuals, individuals younger than 18 years, individuals with disability, single parents, individuals identifying as being from racial and ethnic or language minority groups, crowded housing, and poor transportation facilities, had fewer opportunities to select the highest-quality MA plans. Higher star ratings are associated with receiving care at a higher-quality hospital or nursing home,^4,25,26^ lower rates of hospital readmission,^4^ and voluntary disenrollment.^4,27,28^ If higher star ratings deliver better health outcomes and access, our findings of regional differences in star ratings suggest that such differences could be associated with the observed regional variations in health outcomes and spending in the Medicare program, including among MA enrollees.^29,30^

The highest-rated plans are eligible for the highest-quality bonuses and the highest rebate payments to fund supplemental benefits. With the current regulatory flexibilities, MA plans are now allowed to offer supplemental benefits to address social determinants of health, such as transportation, groceries, meals, home modifications and safety devices, and air purifiers.^31,32,33^ Systematically lower star ratings of plans in socially vulnerable counties imply lower supplemental benefit funding for these plans, which is likely to disadvantage the enrollees who could benefit the most from such innovative benefits. Prior research has documented that MA plans are slow to adopt these newer benefits because of operational, logistic, and funding challenges.^32,34^ Offering these benefits requires plans to partner with third-party organizations and to develop extensive infrastructure that meets Medicare requirements.^32,34^ To scale up the implementation and realize any anticipated positive equity impact of these regulatory flexibilities for MA plans in designing their supplemental benefits, the CMS needs to assess whether enrollees who need these services most are systematically enrolled in plans with poor funding support.

Our finding that plans serving Medicare beneficiaries living in vulnerable regions are less likely to achieve the highest star ratings could inform 2 policy solutions. The first is a modification of the quality bonus payments associated with star rating. Currently, the CMS adjusts the county-level benchmark for traditional Medicare spending quartile, urbanicity, and MA penetration^7^ but not for the multidimensional nature of social vulnerability, which can impact a plan’s performance on star rating measures. To improve equity, the CMS should consider policies that reward plans serving vulnerable regions beyond the risk-adjusted capitation payment that controls for certain sociodemographic characteristics of individual enrollees. To support plans serving socially vulnerable regions, county-level increases in benchmarks could be considered. The CMS plans to begin adjusting the star rating measures in 2027 using a health equity index, which will penalize plans that fail to provide equitable experience and outcomes to all their enrollees.^15,35^ Along with this critical step toward incentivizing equity, accounting for regional vulnerabilities in plan payments could also incentivize plans serving vulnerable regions and avoid higher payments to plans with higher quality ratings that do not serve vulnerable beneficiaries.

The second policy solution could be the implementation of plan-level star ratings. Currently, star ratings are published at the level of contracts and are applied to all plans within a contract. At times, plans in a contract could be serving different counties and, thus, populations with different social vulnerabilities. There is an increasing consensus among stakeholders, including the Medicare Payment Advisory Commission, to rate the quality of individual plans using fewer population-based measures that evaluate the quality at the local market level and to consider social risk factors.^36^ Such a revamping of the star rating system has the potential to align quality bonuses for plans with their regional context and thereby make plans more accountable to their local areas.

### Limitations

Our findings should be interpreted in the light of the following limitations. First, our analysis was limited to regional variations in star ratings. Examining associations between regional vulnerability, star rating, and other plan characteristics, such as premiums and out-of-pocket spending, could be informative. Second, while we studied the association of the overall star rating with different types of vulnerabilities, we did not examine the specific star rating measures separately. Future research that identifies which star rating measures are rated low in socially vulnerable regions can inform policies for potentially modifying the star rating measures or their weights for plans serving vulnerable regions. Third, the goal of this study was to highlight regional variations, and therefore, we did not consider the beneficiary-level mix of plans. Fourth, we used the most recent version of SVI that was currently available when this study was conducted. Any changes in the social vulnerability of counties after 2020 are not reflected in this analysis. Fifth, we chose SVI as the measure of vulnerability because it has been widely used in health services and policy research and allowed us to evaluate star ratings across different types of social vulnerabilities and examine differences at the county level, which is the unit of MA plan contracting and payments. However, SVI data come from survey responses (which could be subject to response bias) and provide equal weight to each measure, unlike the recently developed neighborhood disadvantage measure, the Social Vulnerability Metric. The Social Vulnerability Metric does not include racial and ethnic composition and is only available as a single composite score, but it was shown to outperform the SVI in predicting age-adjusted all-cause mortality.^37^

## Conclusions

In this cross-sectional study, MA plans offered in highly socially vulnerable counties were found to be more likely to have lower star ratings than those in less vulnerable counties. Such regional differences in star ratings could be creating disparities in plan choices available to Medicare beneficiaries, which could be leading to regional differences in health outcomes, care experiences, and spending of Medicare beneficiaries. An inability of plans operating in highly socially vulnerable markets to achieve the highest star ratings implies lower-quality bonuses and rebate dollars for these plans, limiting their ability to invest in improving care or to offer supplemental benefits that can address social determinants of health, despite possibly a high need for these benefits among their enrollees. To achieve equity goals, CMS policies should consider local-market social vulnerability when assigning plan star ratings or should systematically raise the benchmarks for vulnerable counties to support plans serving communities with fewer resources.

## Notes

Supplement 1. **eFigure 1**. Study Sample Selection Flowchart for 2023 **eFigure 2.** Map of US County-Level Average Medicare Advantage Plan Star Rating and Social Vulnerability Quintile Rank Based on the Composite Social Vulnerability Index in 2022 and 2023 **eTable 1**. County-Level MA Plan Characteristics by Quintiles of Social Vulnerability in 2022 and 2023 **eTable 2.** Star Rating Outcomes by County Vulnerability as Compared to the Least Vulnerable Counties, 2023 **eTable 3.** Average Star Rating Outcomes by County Vulnerability Based on The Overall SVI Quintile Ranking for 2023 **eTable 4.** Marginal Effects of Theme-Specific SVI Quintiles on Star Rating Outcomes, 2023 **eTable 5.** Star Rating Outcomes by County Vulnerability as Compared to the Least Vulnerable Counties, 2022 **eFigure 3.** Star Rating Outcomes Shown by Quintiles of the Overall Social Vulnerability Index Score, 2022 **eAppendix.** Results From Sensitivity Analysis Using Star Rating Data From 2022 **eTable 6.** Average Star Rating Outcomes in 2023 by County Vulnerability Based on the SDI Quintile Ranking

Supplement 2.
Data Sharing Statement
