# Supplementary material for: Medicare Advantage Plan Star Ratings and County Social Vulnerability
Source: JAMA Netw Open. 2024 Jul 23;7(7):e2424089. doi: 10.1001/jamanetworkopen.2024.24089 (PMC11267407; doi:10.1001/jamanetworkopen.2024.24089)
Supplement: Supplement 1. — eFigure 1. Study Sample Selection Flowchart for 2023 eFigure 2. Map of US County-Level Average Medicare Advantage Plan Star Rating and Social Vulnerability Quintile Rank Based on the Composite Social Vulnerability Index in 2022 and 2023 eTable 1. County-Level MA Plan Characteristics by Quintiles of Social Vulnerability in 2022 and 2023 eTable 2. Star Rating Outcomes by County Vulnerability as Compared to the Least Vulnerable Counties, 2023 eTable 3. Average Star Rating Outcomes by County Vulnerability Based on The Overall SVI Quintile Ranking for 2023 eTable 4. Marginal Effects of Theme-Specific SVI Quintiles on Star Rating Outcomes, 2023 eTable 5. Star Rating Outcomes by County Vulnerability as Compared to the Least Vulnerable Counties, 2022 eFigure 3. Star Rating Outcomes Shown by Quintiles of the Overall Social Vulnerability Index Score, 2022 eAppendix. Results From Sensitivity Analysis Using Star Rating Data From 2022 eTable 6. Average Star Rating Outcomes in 2023 by County Vulnerability Based on the SDI Quintile Ranking [file jamanetwopen-e2424089-s001.pdf]

Supplementary Online Content

Gupta A, Silver D, Meyers DJ, Glied S, Pagán JA. Medicare Advantage Plan star ratings and county social vulnerability. *JAMA Netw Open*. 2024;7(7):e2424089. doi:10.1001/jamanetworkopen.2024.24089

eFigure 1. Study Sample Selection Flowchart for 2023

eFigure 2. Map of US County-Level Average Medicare Advantage Plan Star Rating and Social Vulnerability Quintile Rank Based on the Composite Social Vulnerability Index in 2022 and 2023

eTable 1. County-Level MA Plan Characteristics by Quintiles of Social Vulnerability in 2022 and 2023

eTable 2. Star Rating Outcomes by County Vulnerability as Compared to the Least vulnerable Counties, 2023

eTable 3. Average Star Rating Outcomes by County Vulnerability Based on The overall SVI Quintile Ranking for 2023

eTable 4. Marginal Effects of Theme-Specific SVI Quintiles on Star Rating Outcomes, 2023

eTable 5. Star Rating Outcomes by County Vulnerability as Compared to the Least Vulnerable Counties, 2022

eFigure 3. Star Rating Outcomes Shown by Quintiles of the Overall Social Vulnerability Index Score, 2022

eAppendix 1. Results From Sensitivity Analysis Using Star Rating Data From 2022

eTable 6. Average Star Rating Outcomes in 2023 by County Vulnerability Based on the SDI Quintile Ranking

This supplementary material has been provided by the authors to give readers additional information about their work.

© 2024 Gupta A et al. *JAMA Network Open*.

eFigure 2: Study sample selection flowchart for 2023

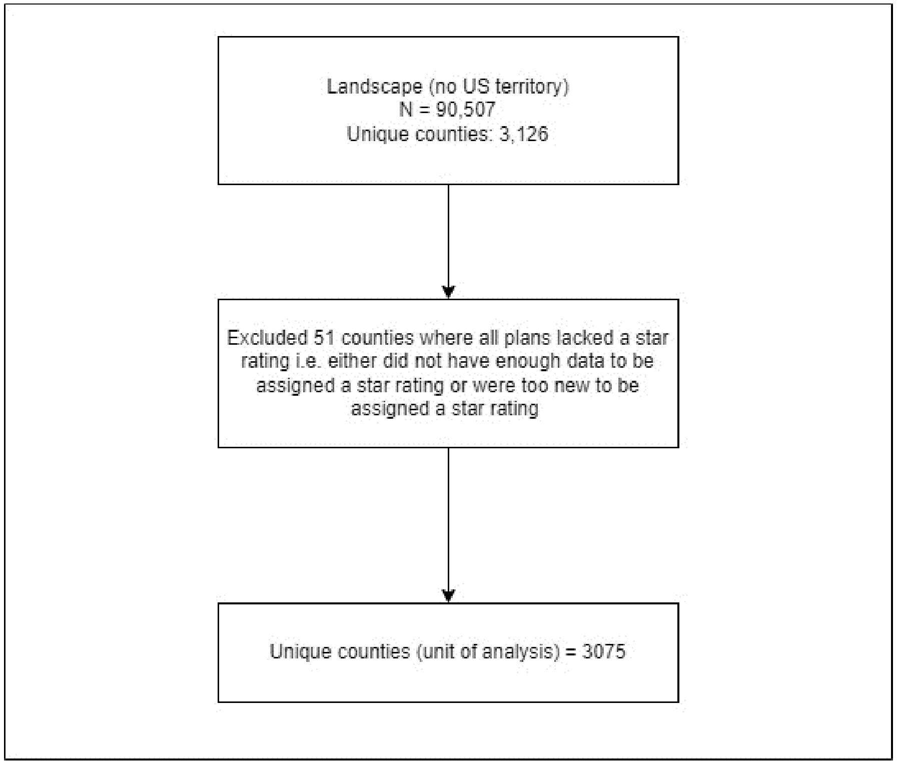

© 2024 Gupta A et al. *JAMA Network Open*.

eFigure 2: Map of US county-level average Medicare Advantage plan star rating and social vulnerability quintile rank based on the composite social vulnerability index in 2022 and 2023

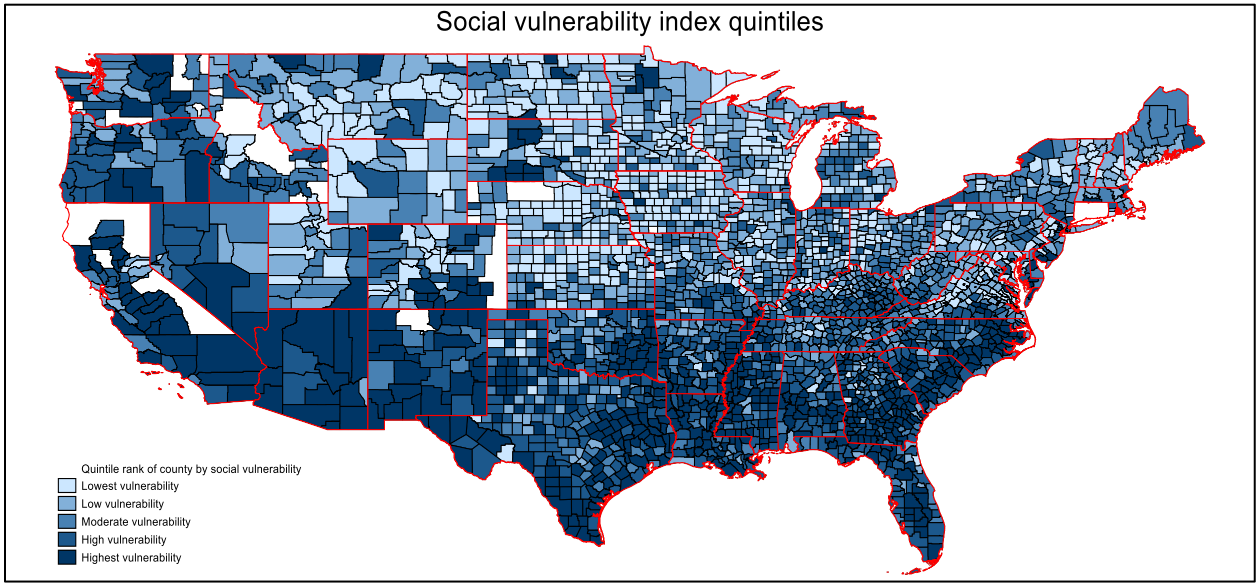

© 2024 Gupta A et al. *JAMA Network Open*.

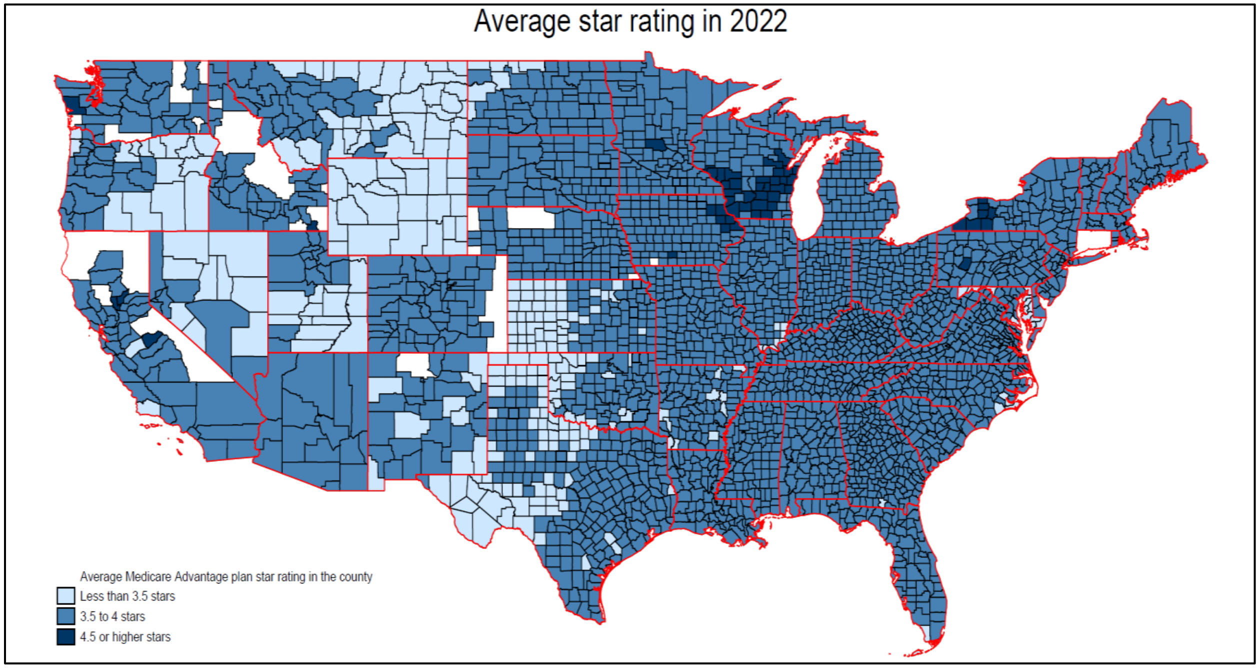

© 2024 Gupta A et al. *JAMA Network Open*.

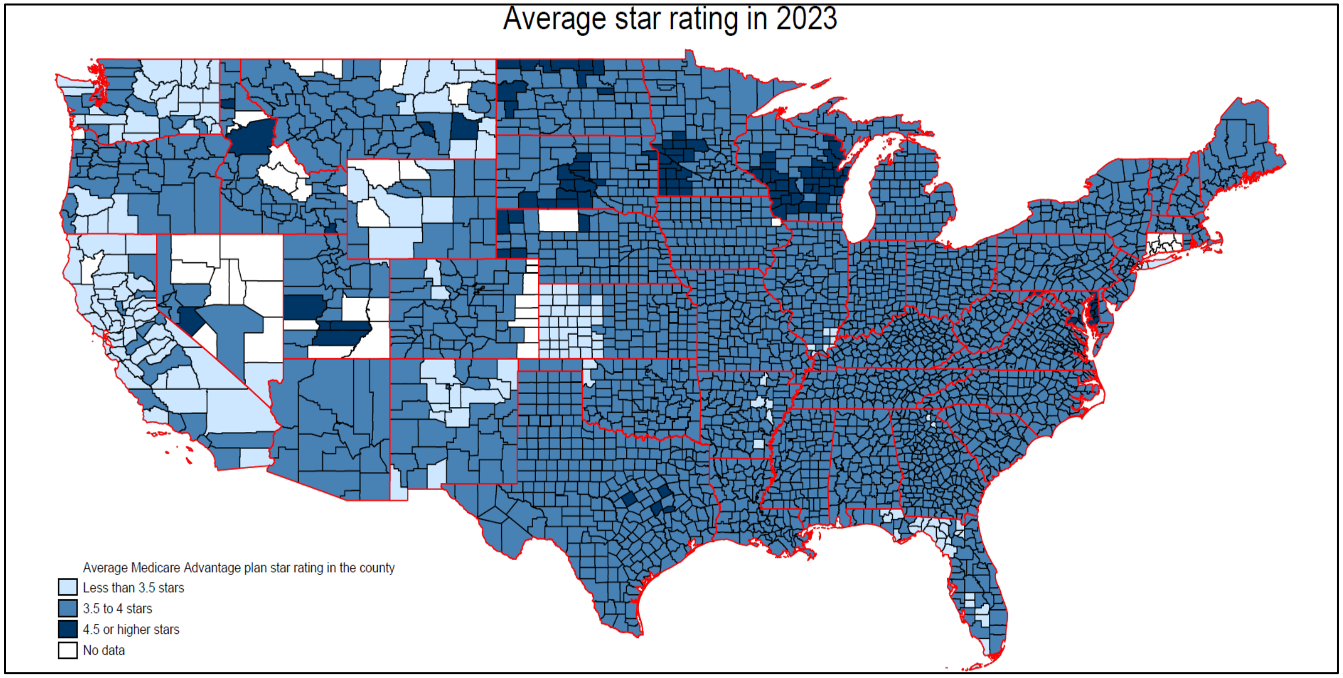

© 2024 Gupta A et al. *JAMA Network Open*.

eTable 2: County-level MA plan characteristics by quintiles of social vulnerability in 2022 and 2023

**County-level outcomes**	**2022 (N = 3,092)**							**2023 (N = 3,075)**						
	**All counties**	**Lowest vulnerability (Q1)**	**Low (Q2)**	**Moderate (Q3)**	**High (Q4)**	**Highest (Q5)**		**All counties**	**Lowest vulnerability (Q1)**	**Low (Q2)**	**Moderate (Q3)**	**High (Q4)**	**Highest (Q5)**	
	**Median (IQR)**	**Median (IQR)**	**Median (IQR)**	**Median (IQR)**	**Median (IQR)**	**Median (IQR)**	**P value**	**Median (IQR)**	**Median (IQR)**	**Median (IQR)**	**Median (IQR)**	**Median (IQR)**	**Median (IQR)**	**P value**
**Number of plans**	23 (15-33)	21 (13-31)	25 (15-34)	25 (17-34)	25 (17-34)	21 (15-29)	<0.001	29 (19-38)	27 (17-36)	30 (20-40)	31 (20-39)	30 (20=-40)	27 (19-35)	<0.001
**Number of plans without sufficient data for star rating**	0 (0-0)	0 (0-0)	0 (0-0)	0 (0-1)	0 (0-1)	0 (0-0)	<0.05	2 (2-3)	2 (1-3)	2 (2-3)	2 (2-3)	2 (2-3)	2 (2-2)	<0.001
**Number of plans too new to be measured for star rating**	2 (0-3)	2 (0-3)	2 (0-3)	2 (0-3)	2 (0-3)	1 (0-3)	0.204	0 (0-3)	0 (0-0)	0 (0-3)	0 (0-2)	0 (0-3)	0 (0-3)	0.053
**Average star rating**	3.9 (3.6-4.1)	4 (3.7-4.2)	3.9 (3.7-4.2)	3.9 (3.7-4.1)	3.8 (3.6-4)	3.7 (3.6-3.9)	<0.001	3.9 (3.7-4.1)	4.1 (3.9-4.3)	4 (3.8-4.2)	3.9 (3.8-4.1)	3.9 (3.7-4.1)	3.8 (3.6-4)	<0.001
**Number of plans rated <3.5 stars**	2 (2-2)	2 (0-2)	2 (0-2)	2 (2-2)	2 (2-3)	2 (2-4)	P<0.001	4 (0-6)	2 (0-4)	3 (0-6)	4 (1-6)	4 (2-7)	4 (2-8)	<0.001
**Number of plans rated 3.5- to 4 stars**	13 (8-18)	11 (7-17)	13 (8-18)	13 (8-18)	13 (9-19)	13 (9-18)	P<0.001	10 (6-15)	8 (4-13)	10 (7-15)	11 (7-16)	11 (7-16)	10 (6-15)	<0.001
**Number of plans rated ≥4.5 stars**	6 (3-14)	6.5 (3-15)	7 (3-16)	7 (3-15)	6 (3-13)	4 (1-9)	P<0.001	13 (7-18)	13 (8-20.5)	13 (7-21)	14 (7-19)	13 (7-17)	11 (7.5-15)	<0.001
**Overall SVI**	0.5 (0.2-0.7)	0.1 (0.05-0.15)	0.3 (0.2-0.3)	0.5 (0.4-0.6)	0.7 (0.6-0.7)	0.9 (0.8-0.9)	P<0.001	0.5 (0.2-0.7)	0.1 (0.05-0.15)	0.3 (0.2-0.3)	0.5 (0.45-0.5)	0.7 (0.6-0.7)	0.90 (0.8-0.95)	<0.001

© 2024 Gupta A et al. *JAMA Network Open*.

eTable 2: Star rating outcomes by county vulnerability as compared to the least vulnerable counties, 2023

County-level outcomes	Average Star Rating		Number of low rated plans		Number of high rated plans		Number of highest rated plans	
	Coefficient (95% CI)[a]	P value	IRR (95% CI)[b]	P value	IRR (95% CI)[b]	P value	IRR (95% CI)[b]	P value
**Overall SVI quintile**								
**Lowest (Q1)**	Ref		Ref		Ref		Ref	
**Low (Q2)**	-0.06 (-0.09 to -0.03)	<0.01	1.31 (1.16 to 1.50)	<0.001	1.26 (1.1 to 1.2)	<0.001	1.02 (0.9 to 1.09)	0.672
**Moderate (Q3)**	-0.12 (-0.15 to -0.08)	<0.001	1.60 (1.41 to 1.82)	<0.001	1.21 (1.12 to 1.30)	<0.001	0.95 (0.90 to 1.03)	0.212
**High (Q4)**	-0.17 (-0.21 to -0.14)	<0.001	1.76 (1.55 to 1.99)	<0.001	1.23 (1.15 to 1.3)	<0.001	0.90 (0.81 to 0.93)	<0.001
**Highest (Q5)**	-0.24 (-0.28 to -0.21)	<0.001	1.81 (1.61 to 2.06)	<0.001	1.1 (1.02 to 1.2)	<0.05	0.75 (0.70 to 0.81)	<0.001
**SES SVI quintile**								
**Lowest (Q1)**	Ref		Ref		Ref		Ref	
**Low (Q2)**	-0.11 (-0.14 to -0.07)	<0.001	1.5 (1.3 to 1.7)	<0.001	1.2 (1.1 to 1.3)	<0.001	0.92 (0.85 to 0.99)	<0.05
**Moderate (Q3)**	-0.15 (-0.19 to -0.12)	<0.001	1.6 (1.4 to 1.8)	<0.001	1.2 (1.1 to 1.4)	<0.001	0.88 (0.81 to 0.94)	<0.01
**High (Q4)**	-0.19 (-0.22 to -0.16)	<0.001	1.8 (1.6 to 2.1)	<0.001	1.2 (1.1 to 1.3)	<0.001	0.83 (0.80 to 0.90)	<0.001
**Highest (Q5)**	-0.26 (-0.29 to -0.23)	<0.001	1.9 (1.7 to 2.3)	<0.001	1.1 (1.0 to 1.2)	<0.05	0.73 (0.70 to 0.79)	<0.001
**Household SVI quintile**								
**Lowest (Q1)**	Ref		Ref		Ref		Ref	
**Low (Q2)**	0.01 (-0.02 to 0.04)	0.566	1.05 (0.9 to 2.0)	0.428	1.1 (1.01 to 1.2)	<0.05	1.1 (1.01 to 1.17)	<0.05

© 2024 Gupta A et al. *JAMA Network Open*.

County-level outcomes	Average Star Rating		Number of low rated plans		Number of high rated plans		Number of highest rated plans	
	Coefficient (95% CI)[a]	P value	IRR (95% CI)[b]	P value	IRR (95% CI)[b]	P value	IRR (95% CI)[b]	P value
**Moderate (Q3)**	-0.07 (-0.1 to 0.03)	<0.001	1.4 (1.2 to 1.5)	<0.001	1.1 (1.03 to 1.2)	<0.01	1.05 (0.9 to 1.13)	0.228
**High (Q4)**	-0.08 (-0.12 to -0.05)	<0.001	1.3 (1.1 to 1.5)	<0.001	1.12 (1.04 to 1.2)	<0.01	0.95 (0.90 to 1.02)	0.269
**Highest (Q5)**	-0.16 (-0.19 to -0.12)	<0.001	1.2 (1.02 to 1.3)	<0.05	0.97 (0.90 to 1.05)	0.489	0.76 (0.70 to 0.82)	<0.001
**Minority SVI quintile**								
**Lowest (Q1)**	Ref		Ref		Ref		Ref	
**Low (Q2)**	-0.02 (-0.06 to 0.01)	0.131	1.1 (0.9 to 1.2)	0.300	0.94 (0.87 to 1.02)	0.127	1.00 (0.93 to 1.08)	0.904
**Moderate (Q3)**	-0.12 (-0.15 to -0.08)	<0.001	1.3 (1.1 to 1.5)	<0.001	0.98 (0.91 to 1.05)	0.567	0.87 (0.80 to 0.93)	<0.001
**High (Q4)**	-0.21 (-0.25 to -0.18)	<0.001	1.6 (1.4 to 1.8)	<0.001	1.05 (0.9 to 1.1)	0.225	0.81 (0.75 to 0.87)	<0.001
**Highest (Q5)**	-0.24 (-0.27 to -0.21)	<0.001	1.8 (1.6 to 2.0)	<0.001	1.03 (0.9 to 1.1)	0.474	0.7 (0.6 to 0.8)	<0.001
**Housing & Transportation SVI quintile**								
**Lowest (Q1)**	Ref		Ref		Ref		Ref	
**Low (Q2)**	-0.02 (-0.05 to 0.01)	0.183	1.2 (1.05 to 1.4)	<0.01	1.1 (1.02 to 1.2)	<0.05	1.1 (1.04 to 1.2)	<0.01
**Moderate (Q3)**	-0.21 (-0.06 to 0.01)	0.227	1.3 (1.1 to 1.5)	<0.001	1.1 (1.03 to 1.3)	<0.01	1.1 (1.02 to 1.2)	<0.05
**High (Q4)**	-0.06 (-0.09 to -0.02)	<0.01	1.4 (1.2 to 1.6)	<0.001	1.2 (1.1 to 1.3)	<0.001	1.1 (1.02 to 1.2)	<0.01
**Highest (Q5)**	-0.16 (-0.19 to -0.12)	<0.001	1.6 (1.4 to 1.8)	<0.001	1.2 (1.1 to 1.3)	<0.001	0.90 (0.8 to 0.96)	<0.01

© 2024 Gupta A et al. *JAMA Network Open*.

County-level outcomes	Average Star Rating		Number of low rated plans		Number of high rated plans		Number of highest rated plans	
	Coefficient (95% CI)[a]	P value	IRR (95% CI)[b]	P value	IRR (95% CI)[b]	P value	IRR (95% CI)[b]	P value

[a]Estimates are coefficients derived from bivariable linear regression models for average star rating, [b]Estimates are coefficients derived from bivariable negative binomial models for the number of low-, high- and highest-rated plans. Abbreviations: IRR: Incident Rate Ratio; SVI: Social Vulnerability Index

© 2024 Gupta A et al. *JAMA Network Open*.

eTable 3: Average star rating outcomes by county vulnerability based on the overall SVI quintile ranking for 2023

County-level outcomes	Average Star Rating		Number of low rated plans		Number of high rated plans		Number of highest rated plans	
	Average (95% CI)[a]	P value	Average (95% CI)[a]	P value	Average (95% CI)[a]	P value	Average (95% CI)[a]	P value
**Overall SVI**								
**Lowest**	4.05 (4.02 to 4.07)	<0.001	2.79 (2.53 to 3.05)	<0.001	9.68 (9.17 to 10.19)	<0.001	15.42 (14.61 to 16.23)	<0.001
**Low**	3.99 (3.96 to 4.01)	<0.001	3.67 (3.33 to 3.99)	<0.001	11.23 (10.65 to 11.81)	<0.001	15.7 (14.8 to 16.5)	<0.001
**Moderate**	3.93 (3.90 to 3.95)	<0.001	4.47 (4.07 to 4.86)	<0.001	11.72 (11.11 to 12.33)	<0.001	14.7 (13.9 to 15.5)	<0.001
**High**	3.87 (3.85 to 3.89)	<0.001	4.9 (4.48 to 5.34)	<0.001	11.97 (11.3 to 12.6)	<0.001	13.4 (12.7 to 14.1)	<0.001
**Highest**	3.80 (3.77 to 3.83)	<0.001	5.06 (4.62 to 5.5)	<0.001	10.6 (10.1 to 11.2)	<0.001	11.64 (11.01 to 12.32)	<0.001

[a]Estimates shown are margins calculated from regression models for each star rating outcome and represent the average star rating outcome at each overall SVI quintile. Abbreviations: SVI: Social Vulnerability Index.

© 2024 Gupta A et al. *JAMA Network Open*.

eTable 4: Marginal effects of theme-specific SVI quintiles on star rating outcomes, 2023

County-level outcomes	Average Star Rating		Number of low rated plans		Number of high rated plans		Number of highest rated plans	
	dy/dx (95% CI)[a]	P value	dy/dx (95% CI)[b]	P value	dy/dx (95% CI)[b]	P value	dy/dx (95% CI)[b]	P value
**SES SVI**								
**Lowest**	Ref		Ref		Ref		Ref	
**Low**	-0.11 (-0.14 to -0.07)	<0.001	1.4 (0.96 to 1.82)	<0.001	1.7 (0.9 to 2.4)	<0.001	-1.3 (-2.4 to -0.1)	<0.05
**Moderate**	-0.15 (-0.19 to -0.12)	<0.001	1.6 (1.2 to 2.1)	<0.001	2.3 (1.5 to 3.1)	<0.001	-2.0 (-3.1 to -0.8)	<0.01
**High**	-0.19 (-0.22 to -0.16)	<0.001	2.1 (1.7 to 2.6)	<0.001	1.8 (1.1 to 2.6)	<0.001	-2.7 (-3.7 to -1.5)	<0.001
**Highest**	-0.26 (-0.29 to -0.23)	<0.001	2.6 (2.1 to 3.1)	<0.001	0.9 (0.2 to 1.7)	<0.05	-4.3 (-5.4 to -3.2)	<0.001
**Household SVI**								
**Lowest**	Ref		Ref		Ref		Ref	
**Low**	0.01 (-0.02 to 0.04)	0.566	0.19 (0.9 to 2.0)	0.428	0.9 (0.1 to 1.7)	<0.05	1.3 (0.1 to 2.4)	<0.05
**Moderate**	-0.07 (-0.1 to 0.03)	<0.001	1.2 (0.7 to 1.7)	<0.001	1.2 (0.4 to 2.0)	<0.01	0.7 (-0.4 to 1.8)	0.228
**High**	-0.08 (-0.12 to -0.05)	<0.001	0.9 (0.5 to 1.5)	<0.001	1.2 (0.4 to 2.1)	<0.01	-0.7 (-1.8 to 0.3)	0.169
**Highest**	-0.16 (-0.19 to -0.12)	<0.001	0.6 (0.1 to 1.1)	<0.05	-0.3 (-1.0 to 0.5)	0.489	-3.5 (-4.4 to -2.5)	<0.001
**Minority SVI**								
**Lowest**	Ref		Ref		Ref		Ref	
**Low**	-0.02 (-0.06 to 0.01)	0.131	0.2 (-0.2 to 0.6)	0.300	-0.6 (-1.4 to 0.2)	0.127	0.1 (-1.1 to 1.2)	0.904
**Moderate**	-0.12 (-0.15 to -0.08)	<0.001	0.9 (0.5 to 1.3)	<0.001	-0.2 (-1.0 to 0.6)	0.567	-2.1 (-3.2 to -1.1)	<0.001

© 2024 Gupta A et al. *JAMA Network Open*.

County-level outcomes	Average Star Rating		Number of low rated plans		Number of high rated plans		Number of highest rated plans	
	dy/dx (95% CI)[a]	P value	dy/dx (95% CI)[b]	P value	dy/dx (95% CI)[b]	P value	dy/dx (95% CI)[b]	P value
**High**	-0.21 (-0.25 to -0.18)	<0.001	2.0 (1.5 to 2.5)	<0.001	0.5 (-0.3 to 1.4)	0.225	-3.1 (-4.1 to -2.0)	<0.001
**Highest**	-0.24 (-0.27 to -0.21)	<0.001	2.4 (1.9 to 3.0)	<0.001	0.3 (-0.5 to 1.1)	0.474	-4.4 (-5.5 to -3.4)	<0.001
**Housing & Transportation SVI**								
**Lowest**	Ref		Ref		Ref		Ref	
**Low**	-0.02 (-0.05 to 0.01)	0.183	0.6 (0.2 to 1.1)	<0.01	0.99 (0.3 to 1.8)	<0.05	1.6 (0.6 to 2.7)	<0.01
**Moderate**	-0.21 (-0.06 to 0.01)	0.227	0.9 (0.5 to 1.4)	<0.001	1.04 (0.27 to 1.8)	<0.01	1.4 (0.3 to 2.5)	<0.05
**High**	-0.06 (-0.09 to -0.02)	<0.01	1.2 (0.7 to 1.7)	<0.001	1.8 (1.0 to 2.6)	<0.001	1.4 (0.3 to 2.5)	<0.01
**Highest**	-0.16 (-0.19 to -0.12)	<0.001	2.0 (1.4 to 2.5)	<0.001	1.7 (0.9 to 2.5)	<0.001	-1.4 (-2.4 to -0.4)	<0.01

[a]Estimates are marginal effects derived from bivariable linear regression models for average star rating. Marginal effects from bivariable linear regression models are same as coefficients, representing difference in average star rating comparing SVI quintile 2 to 5 with quintile 1.
[b]Estimates shown are marginal effects derived from bivariable negative binomial models for the number of low-, high- and highest-rated plans. Abbreviations: IRR: Incident Rate Ratio; SVI: Social Vulnerability Index.

© 2024 Gupta A et al. *JAMA Network Open*.

eTable 5: Star rating outcomes by county vulnerability as compared to the least vulnerable counties, 2022

County-level outcomes	Average Star Rating		Number of low rated plans		Number of high rated plans		Number of highest rated plans	
	**Coefficient (95% CI)[a]**	**P value**	**IRR (95% CI)[b]**	**P value**	**IRR (95% CI)[b]**	**P value**	**IRR (95% CI)[b]**	**P value**
**Overall SVI quintile**								
**Lowest (Q1)**	Ref		Ref		Ref		Ref	
**Low (Q2)**	-0.02 (-0.68 to 0.27)	0.402	1.1 (1.05 to 1.27)	<0.01	1.1 (1.03 to 1.2)	<0.01	1.1 (0.9 to 1.2)	0.141
**Moderate (Q3)**	-0.08 (-0.13 to -0.04	<0.01	1.3 (1.2 to1.5)	<0.001	1.1 (1.04 to 1.2)	<0.01	1.1 (0.9 to 1.2)	0.239
**High (Q4)**	-0.13 (-0.17 to -0.08)	<0.001	1.6 (1.4 to 1.7)	<0.001	1.2 (1.2 to 1.3)	<0.001	0.9 (0.8 to 1.0)	0.110
**Highest (Q5)**	-0.23 (-0.28 to -0.18)	<0.001	1.7 (1.5 to 1.8)	<0.001	1.2 (1.1 to 1.2)	<0.001	0.6 (0.5 to 0.7)	<0.001
**SES SVI quintile**								
**Lowest (Q1)**	Ref		Ref		Ref		Ref	
**Low (Q2)**	-0.03 (-0.08 to 0.14)	0.166	1.1 (1.02 to 1.2)	<0.05	0.9 (0.08 to 1.8)	<0.05	1.1 (0.9 to 1.2)	0.104
**Moderate (Q3)**	-0.11 (-0.16 to -0.6)	<0.001	1.3 (1.2 to 1.4)	<0.001	1.7 (0.8 to 2.6)	<0.001	0.9 (0.8 to 1.1)	0.505
**High (Q4)**	-0.13 (-0.18 to -0.08)	<0.001	1.5 (1.3 to 1.6)	<0.001	2.3 (1.4 to 3.2)	<0.001	0.8 (0.7 to 0.9)	<0.01
**Highest (Q5)**	-0.21 (-0.26 to -0.17)	<0.001	1.6 (1.5 to 1.7)	<0.001	2.6 (1.7 to 3.5)	<0.001	0.6 (0.5 to 0.7)	<0.001
**Household SVI quintile**								
**Lowest (Q1)**	Ref		Ref		Ref		Ref	
**Low (Q2)**	0.02 (-0.03 to 0.07)	0.411	1.2 (1.1 to 1.3)	<0.001	1.1 (1.02 to 1.2)	<0.01	1.1 (0.9 to 1.2)	0.133
**Moderate (Q3)**	-0.02 (-0.07 to 0.02)	0.317	1.4 (1.2 to 1.5)	<0.001	1.2 (1.06 to 1.2)	<0.001	1.04 (0.9 to 1.2)	0.418
**High (Q4)**	-0.12 (-0.17 to -0.07)	<0.001	1.5 (1.3 to 1.6)	<0.001	1.1 (1.1 to 1.2)	<0.001	0.8 (0.7 to 0.9)	<0.01

© 2024 Gupta A et al. *JAMA Network Open*.

County-level outcomes	Average Star Rating		Number of low rated plans		Number of high rated plans		Number of highest rated plans	
	Coefficient (95% CI)[a]	P value	IRR (95% CI)[b]	P value	IRR (95% CI)[b]	P value	IRR (95% CI)[b]	P value
**Highest (Q5)**	-0.18 (-0.22 to -0.13)	<0.001	1.5 (1.3 to 1.6)	<0.001	1.04 (0.9 to 1.1)	0.222	0.6 (0.5 to 0.7)	<0.001
**Minority SVI quintile**								
**Lowest (Q1)**	Ref		Ref		Ref		Ref	
**Low (Q2)**	-0.07 (-0.12 to -0.03)	<0.01	1.1 (1.02 to 1.2)	<0.05	-0.9 (-1.8 to -0.06)	<0.05	1.1 (0.9 to 1.2)	0.234
**Moderate (Q3)**	-0.13 (-0.18 to -0.08)	<0.001	1.2 (1.2 to 1.3)	<0.001	-0.9 (-1.8 to -0.2)	<0.05	0.9 (0.8 to 1.1)	0.697
**High (Q4)**	-0.23 (-0.27 to -0.18)	<0.001	1.5 (1.3 to 1.6)	<0.001	0.6 (-0.3 to 1.5)	0.229	0.8 (0.7 to 0.9)	<0.01
**Highest (Q5)**	-0.30 (-0.35 to -0.26)	<0.001	1.6 (1.5 to 1.8)	<0.001	0.8 (-0.15 to 1.7)	0.101	0.7 (0.6 to 0.7)	<0.001
**Housing & Transportation SVI quintile**								
**Lowest (Q1)**	Ref		Ref		Ref		Ref	
**Low (Q2)**	0.05 (0.003 to 0.09)	<0.05	1.1 (0.9 to 1.2)	0.072	1.1 (1.02 to 1.2)	<0.05	1.2 (1.1 to 1.4)	<0.001
**Moderate (Q3)**	0.04 (-0.002 to 0.09)	0.066	1.2 (1.1 to 1.3)	<0.001	1.05 (0.9 to 1.1)	0.188	1.3 (1.2 to 1.5)	<0.001
**High (Q4)**	0.03 (-0.01 to 0.08)	0.177	1.2 (1.1 to 1.3)	<0.01	1.1 (1.0 to 1.2)	<0.001	1.3 (1.2 to 1.5)	<0.001
**Highest (Q5)**	-0.09 (-0.14 to -0.05)	<0.001	1.4 (1.3 to 1.6)	<0.001	1.1 (1.0 to 1.2)	<0.01	0.9 (0.8 to 1.1)	0.469

[a]Estimates are coefficients derived from bivariable linear regression models for average star rating, [b]Estimates are coefficients derived from bivariable negative binomial models for the number of low-, high- and highest-rated plans. Abbreviations: IRR: Incident Rate Ratio; SVI: Social Vulnerability Index

© 2024 Gupta A et al. *JAMA Network Open*.

eFigure 3: Star Rating Outcomes shown by quintiles of the Overall Social Vulnerability Index Score, 2022

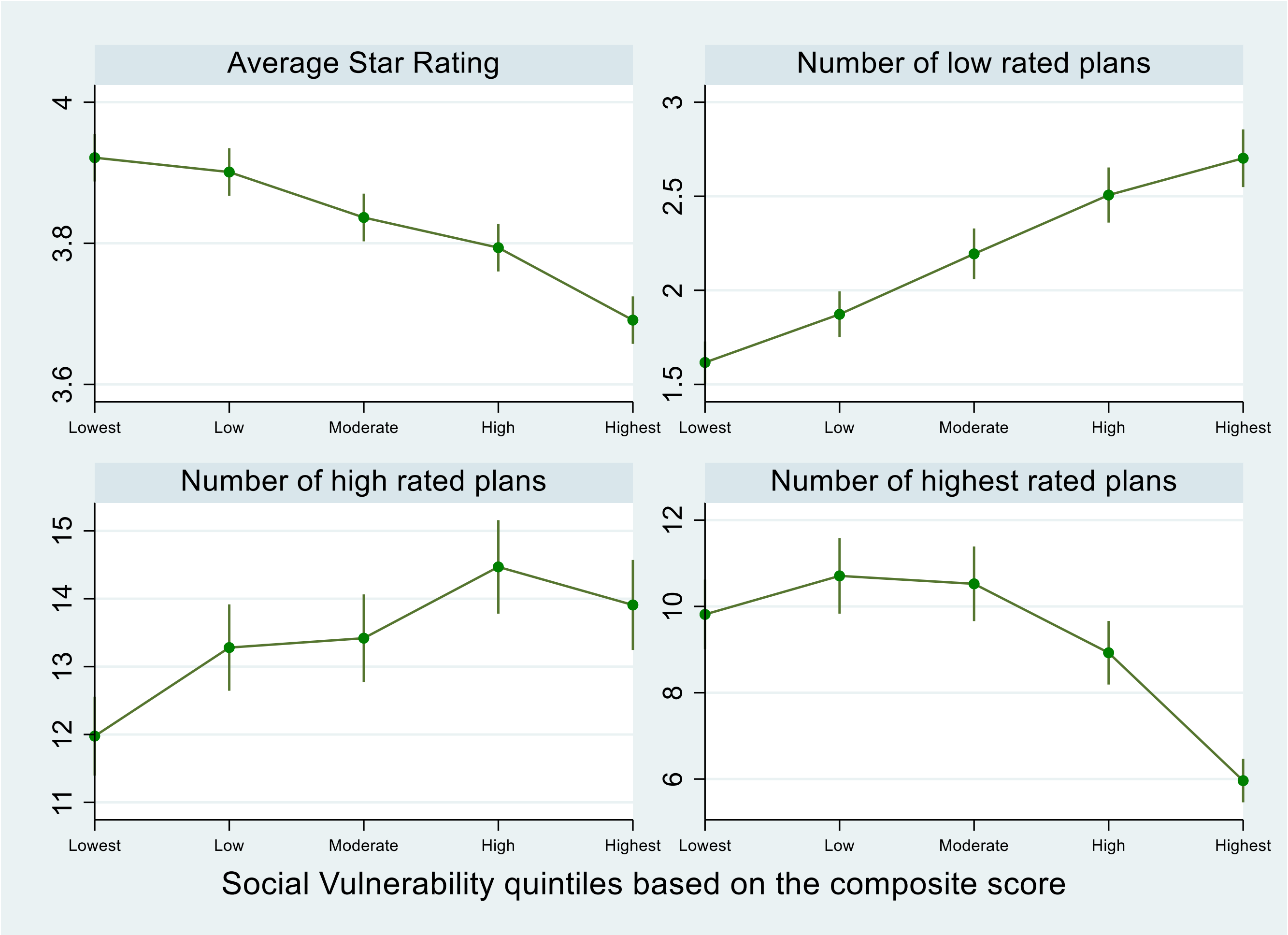

Estimates shown are average outcomes (margins) at each quintile of the composite social vulnerability index score

© 2024 Gupta A et al. *JAMA Network Open*.

eAppendix 1: Results from sensitivity analysis using star rating data from 2022

We examined 3,092 counties in 2022. The median number of plans per county was 23 (IQR: 15-33). After excluding plans lacking sufficient data to measure the star rating and plans that were too new to be assigned a star rating (**eTable 1** shows the number of such plans across SVI quintiles), among plans with a star rating, the median county-level average star rating in 2022 was 3.9 (IQR: 3.6, 4.1). The median county-level average star rating was 4 (3.7-4.2) in Q1 counties and 3.7 (3.6-3.8) in Q5 counties (P<0.001) **(eTable 1).** The median number of highest-rated plans in a county was highest for Q1 and lowest for Q5 counties (p<0.001) (**eTable 1)**. However, the median number of low rated plans in a county was lowest for Q1 counties and highest for Q5 counties (p<0.001) (**eTable 1**).

In the regression models for 2022, the average star rating in Q5 counties based on the composite SVI score was 0.23 points less (-0.23; 95% CI: -0.28 to -0.18; P<0.001), 0.13 less in Q4 counties (-0.13; 95% CI: -0.17 to -0.08; P<0.001), and 0.08 less in Q3 counties (-0.08; 95% CI: -0.13 to -0.04' P<0.01) compared to Q1 counties. The number of low-rated (IRR: 1.7; 95% CI: 1.5 to 1.8; P<0.001) and high-rated plans (IRR: 1.2; 95% CI: 1.1 to 1.2; P<0.001) was higher in Q5 counties than in Q1 counties. However, the number of highest-rated plans was lower (IRR: 0.6; 95% CI: 0.5 to 0.7; P<0.001) in Q5 counties than in Q1 counties (**eFigure 3** and **eTable 5)**

For each theme, the average star rating was lower, the number of low- and high-rated plans was higher, and the number of highest-rated plans was lower in Q5 vs. Q1 counties, except for no difference in the number of high-rated plans for themes 2 and 3, and for the number of highest-rated plans for theme 4 (**eTable 5)**

© 2024 Gupta A et al. *JAMA Network Open*.

eTable 6: Average star rating outcomes in 2023 by county vulnerability based on the SDI quintile ranking

County-level outcomes	Average Star Rating		Number of low rated plans		Number of high rated plans		Number of highest rated plans	
	**Average (95% CI)[a]**	**P value**	**IRR (95% CI)[a]**	**P value**	**Average (95% CI)[a]**	**P value**	**Average (95% CI)[a]**	**P value**
**Overall SDI**								
**Lowest**	Ref		Ref		Ref		Ref	
**Low**	-0.05 (-0.08 to -0.11)	0.009	1.2 (1.1 to 1.4)	0.005	1.05 (0.97 to 1.14)	0.161	0.98 (0.92 to 1.01)	0.766
**Moderate**	-.15 (-0.13 to -0.06))	<0.001	1.47 (1.30 to 1.67)	<0.001	1.21 (1.12 to 1.31)	<0.001	0.94 (0.88 to 1.02)	0.132
**High**	-0.14 (-0.2 to -0.11)	<0.001	1.48 (1.31 to 1.68)	<0.001	1.17 (1.09 to 1.26)	<0.001	0.88 (0.81 to 0.94)	0.001
**Highest**	-0.21 (-0.15 to -0.18)	<0.001	1.56 (1.38 to 1.77)	<0.001	1.18 (1.01 to 1.17)	0.023	0.76 (0.70 to 0.82)	<0.001

[a]Estimates shown are margins calculated from regression models for each star rating outcome and represent the average star rating outcome at each overall SDI quintile. Abbreviations: SDI: Social Deprivation Index.

© 2024 Gupta A et al. *JAMA Network Open*.
