# Supplementary material for: Medicare Advantage Plan Star Ratings and County Social Vulnerability
Source: JAMA Netw Open. 2024 Jul 23;7(7):e2424089. doi: 10.1001/jamanetworkopen.2024.24089 (PMC11267407; doi:10.1001/jamanetworkopen.2024.24089)
Supplement: Supplement 2. — Data Sharing Statement [file jamanetwopen-e2424089-s002.pdf]

Data Sharing Statement

Gupta. Medicare Advantage Plan Star Ratings and County Social Vulnerability. *JAMA Netw Open*. Published July 23, 2024. doi:10.1001/jamanetworkopen.2024.24089

Data
Data available: Yes
Data types: Data (not involving human participants), Data dictionary
How to access data: The data and the data dictionary used for this study are publicly available through CMS web site (https://www.cms.gov/medicare/health-drug-plans/part-c-d-performance-data) and CDC web site (https://www.atsdr.cdc.gov/placeandhealth/svi/index.html).
When available: With publication

Supporting Documents
Document types: None

Additional Information
Who can access the data: Statistical code for this study can be made available upon request which can be made to the corresponding author, Avni Gupta at ag7452@nyu.edu.
Types of analyses: Data is publicly available and the indicated uses of the data are explained at the web sites.
Mechanisms of data availability: Data are publicly available.
